# Improving HIV assisted partner services outcomes by eliciting additional partners after the initial encounter

**DOI:** 10.1371/journal.pgph.0004406

**Published:** 2026-02-03

**Authors:** George Otieno, Sarah Masyuko, Unmesha Roy Paladhi, Edward Kariithi, Monisha Sharma, Hanley Kingston, Harison Lagat, David A. Katz, Beatrice Wamuti, Paul Macharia, Rose Bosire, Mary Mugambi, Bryan J. Weiner, Carey Farquhar

**Affiliations:** 1 PATH-Kenya, Kisumu, Kenya; 2 Department of Global Health, University of Washington, Seattle, Washington, United States of America; 3 Ministry of Health, Nairobi, Kenya; 4 Brown Global Alliance for Infant and Maternal Health Research, Brown University, Providence, Rhode Island, United States of America; 5 Department of Pediatrics, Warren Alpert Medical School, Brown University, Providence, Rhode Island, United States of America; 6 Institute of Public Health Genetics, University of Washington, Seattle, Washington, United States of America; 7 School of Nursing, University of Washington, Seattle, Washington, United States of America; 8 Kenyatta National Hospital, Nairobi, Kenya; 9 Strathmore University, Nairobi, Kenya; 10 Centre for Public Health Research, Kenya Medical Research Institute (KEMRI), Nairobi, Kenya; 11 Department of Epidemiology, University of Washington, Seattle, Washington, United States of America; 12 Department of Medicine, University of Washington, Seattle, Washington, United States of America; University of Embu, KENYA

## Abstract

Most assisted partner services (APS) programs elicit partners at the time of HIV diagnosis when index clients may be reluctant to name all partners. Little is known about the benefits of ongoing partner elicitation after the initial visit. We utilized data collected in an APS implementation study across 31 facilities in western Kenya from August 2019 to June 2022. HIV testing service providers offered APS to consenting female index clients and asked them to name their male partners both at initial diagnosis and during follow-up clinic visits for 12 months. Partners were traced and offered HIV testing. Using multivariable Poisson Generalized Estimated Equation models, we compared characteristics of index clients who did and did not name additional partners and assessed HIV diagnoses and characteristics of partners named during initial versus follow-up visits. The 872 female index clients who accepted APS named 3461 male partners, of whom 2920 (84%) were successfully contacted and HIV tested. Of 1819 male partners named at the initial visit, 430 (23.6%) were previously diagnosed and 90 (4.9%) were newly diagnosed with HIV. Of 1101 male partners named at follow-up visits, 335 (30.4%) were previously diagnosed and 193 (17.5%) were newly diagnosed with HIV. Among partners tested, those named at follow-up visits were 3.9 times more likely to be newly diagnosed with HIV than those named at the initial visit (Relative Risk = 3.88, 95%CI = 3.00–4.98) and were more likely to report behaviors associated with HIV transmission, including having sex with >1 partner (p < 0.001) and with a partner at risk of HIV or with unknown HIV status (p = 0.01). Continuing partner elicitation for APS for 12 months after the initial visit was associated with a higher likelihood of identifying male partners at increased HIV risk compared to those initially named and increased the number of new HIV diagnoses.

## Introduction

HIV remains a significant cause of morbidity globally, with 38 million persons living with HIV and 1.7 million new diagnoses in 2019, with over 40% of those newly diagnosed with HIV residing in Eastern and Southern Africa [1]. Suboptimal knowledge of HIV status is a barrier to epidemic control in sub-Saharan Africa [2,3]. Assisted partner services (APS), which provides exposure notification and HIV testing services to partners of persons diagnosed with HIV (index clients), is an effective and efficient strategy to identify people living with HIV (PLWH), particularly those who are undiagnosed [4,5]. APS is recommended by the World Health Organization (WHO) as part of routine HIV services, and guidelines state that index clients should be offered APS at the time of diagnosis and APS can be repeated during subsequent interactions with providers. However, most APS programs elicit sexual partners only at the initial visit and some index clients may not be ready and comfortable enough to disclose their partners following a new HIV diagnosis.

In the APS Scale-up Study in western Kenya [6], we sought to determine if continuing APS after the initial visit and eliciting male partners from female index clients for 12 months after diagnosis, reached partners with different characteristics and behaviors and increased new HIV diagnoses. The APS Scale-up Study focused on male partners because APS may be especially useful for reaching men, who tend to face unique barriers to HIV testing, including stigma, hesitancy, and lack of male-focused points of care [7,8].

## Methods

### Study design

We conducted a secondary data analysis using data from a hybrid type-2 implementation science study (APS Scale-up Study) conducted in Kisumu and Homa Bay counties in western Kenya, a high HIV prevalence region [7,9], between 1^st^ August 2019 and 27^th^ June 2022. The 31 study sites included a mix of low- and high-volume health facilities ranging from primary to tertiary care. HIV testing services (HTS) were provided in facility and community settings by HTS providers. This study leveraged existing infrastructure of government facilities supported by the U.S. Agency for International Development (USAID)/The U.S. President’s Emergency Plan for AIDS Relief (PEPFAR)-funded program, Afya Ziwani, in collaboration with county and sub-county health management teams in both counties. The implementation team included collaborators from the University of Washington, a non-governmental organization PATH, and the Kenya Ministry of Health [6].

### Study population and procedures

For this secondary analysis, we included index participants were (1) female, (2) testing HIV positive and not in care or on treatment, (3) aged ≥18 years or emancipated minor (girls aged ≥15 years who are married; pregnant; or have had a sexually transmitted infection, including HIV and hence can self-consent), (4) willing to participate in the study, and (5) willing to provide contact information of ≥1 sex partner within the last 3 years and 6) enrolled between August 2019 and March 2020 at a study site. Exclusion criteria were <15 years old, pregnant, or at high risk of intimate partner violence (IPV). Female index clients who were eligible and provided written informed consent were offered APS by HTS providers and asked to provide names and contact information for their male partners from the last 3 years. These were recorded in the standard Ministry of Health APS registers and collected for the study using tablets on the Open Data Kit platform. Index clients were invited to attend follow-up visits at six weeks and three, six, and twelve months after diagnosis to assess linkage to care, including initiation of antiretroviral therapy (ART) or pre-exposure prophylaxis (PrEP), as well as adverse events including IPV or relationship dissolution. At each follow-up visit, HTS providers also offered APS, asking about male sexual partners not mentioned previously and eliciting their names and contact information.

Male partners were eligible for HIV testing via APS if they were ≥18 years old. Eligible male partners were contacted, notified of their possible HIV exposure, and offered HIV testing at either a clinic or through community testing. After obtained informed consent, surveys were used to collect sociodemographic characteristics, behavioral information, HIV testing history, and other data from female index clients and male partners at enrollment [6].

### Human subjects approvals

The study was approved by the Kenyatta National Hospital/University of Nairobi Ethics and Research Committee (P465/052017), University of Washington Institutional Review Board (STUDY00002420), and PATH Institutional Review Board.

### Statistical analyses

We summarized demographics, HIV testing history, sexual and drug use behaviors and partner outcomes for female index clients and male partners. We used univariate and multivariate Poisson Generalized Estimated Equation models with an exchangeable correlation structure and robust standard errors to assess the association between the above characteristics and three outcomes: 1) naming additional partners at a follow-up visit among female index clients (vs. only naming partners at initial visit); 2) being initially named or named at a follow-up visit among male partners; and 3) receiving a new HIV diagnosis among partners who were named after the first visit. Age, county, and behaviors associated with risk of HIV transmission in the last 12 months (with total count >10) were included *a priori* in a multivariate model. In addition, all other variables with p < 0.10 in the univariate model were included in the multivariate model (with the exception of partner outcomes in the index analysis). Because participants with HIV diagnoses before study enrollment were not asked about behaviors associated with HIV transmission, multivariate analyses are limited to the subset of male partners without a prior diagnosis. Effects with p < 0.05 were considered statistically significant. Missing data was very limited and hence we restricted our analysis to variables with complete data in the regression models. Analyses were conducted in Stata 15.1 [10] and R [11].

## Results

### Female index characteristics

We enrolled 872 female index clients from August 2019 to March 2020, of whom 573 (66%) named additional male sexual partners after the initial visit where they received an HIV diagnosis and were followed up until June 2022. The 12-month follow up rate was 89% for female index clients and 90% for the male partners as reported in the main paper [7]. Index clients had a median age of 28 years (Inter-quartile range [IQR]: 23–34), 645 (74%) had completed either primary or post-primary school, 741 (85%) were employed or self-employed, and 532 (61%) were in monogamous marriages or cohabiting (Table 1). The most frequently mentioned sexual behaviors associated with HIV transmission in the last six months were inconsistent condom use (31%) and not using a condom during last sex (29%).

**Table 1:** Characteristics of female index clients who named vs. did not name additional partners via assisted partner services after their initial encounter.

Variable	Index clients without additional partners N = 299	Index clients with additional partners N = 573	Total N = 872
	n (%) or median (IQR^a^)	n (%) or median (IQR)	n (%) or median (IQR)
**Socio-demographic characteristics**			
Age	28 (23,34)	28 (22,34)	28 (23,34)
Marital status			
Single/Never married	46 (15.4)	111 (19.4)	157 (18.0)
Married (monogamous)/Cohabitating	195 (65.2)	337 (58.8)	532 (61.0)
Married (polygamous)	13 (4.3)	34 (5.9)	47 (5.4)
Divorced/Separated/Widowed	45 (15.1)	91 (15.9)	136 (15.6)
Highest education completed			
Did not complete primary school	86 (28.8)	141 (24.6)	227 (26.0)
Completed primary school	137 (45.8)	285 (49.7)	422 (48.4)
Completed secondary school	76 (25.4)	147 (25.7)	223 (25.6)
Occupation			
Employed/Self-employed	257 (86.0)	484 (84.5)	741 (85.0)
Unemployed	19 (6.4)	45 (7.9)	64 (7.3)
Student	23 (7.7)	44 (7.7)	67 (7.7)
Average monthly household income			
**≤** 87 USD^b^	229 (76.6)	452 (78.9)	681 (78.1)
> 87 USD	70 (23.4)	121 (21.1)	191 (21.9)
County			
Kisumu	210 (70.2)	200 (34.9)	410 (47.0)
Homa Bay	89 (29.8)	373 (65.1)	462 (53.0)
**HIV testing and sexual history**			
Testing modality			
Non-facility based	114 (38.1)	145 (25.3)	259 (29.7)
Facility based	185 (61.9)	428 (74.7)	613 (70.3)
Testing approach			
Individual	288 (96.3)	549 (95.8)	837 (96.0)
Couple	11 (3.7)	24 (4.2)	35 (4.0)
HIV test prior to study enrollment	233 (77.9)	475 (82.9)	708 (81.2)
HIV self-test in last 12 months	27 (9.0)	24 (4.2)	51 (5.8%)
Number of male partners in the last 3 years	3 (2,4)	4 (4,5)	4 (3,5)
**Behaviors associated with HIV transmission in the last 12 months**			
Any	164 (54.8)	283 (49.4)	447 (51.3)
Inconsistent condom use	108 (36.1)	163 (28.4)	271 (31.1)
Did not use condom during last sex	93 (31.1)	162 (28.3)	255 (29.2)
Recent sexually transmitted infection (STI)	6 (2.0)	10 (1.7)	16 (1.8)
Recurrent use of post-exposure prophylaxis (PEP)	0	1 (0.2)	1 (0.1)
Recurrent sex under influence of alcohol/recreational drugs	8 (2.7)	3 (0.5)	11 (1.3)
Sex with more than one partner	68 (22.7)	84 (14.7)	152 (17.4)
Sex partner(s) at high risk for HIV and HIV status unknown	8 (2.7)	14 (2.4)	22 (2.5)
Sex partner(s) were HIV positive	4 (1.3)	9 (1.6)	13 (1.5)
Transactional sex	0	1 (0.2)	1 (0.1)
IDU with shared needles/syringes	0	0	0
**Partner outcomes**			
1 + partners newly diagnosed with HIV	44 (14.7)	220 (38.4)	264 (30.3)

^a^IQR: interquartile range.

^b^USD: United States dollar (data was collected in Kenya shillings, with about Kenya shillings 10,000 exchanging for 87 USD).

Female index clients from Homa Bay (vs. Kisumu) were more likely to name additional male partners (Relative Risk [RR]: 1.66, 95% Confidence Interval [CI]: 1.33-2.05; adjusted Relative Risk [aRR]: 1.25, 95% CI: 0.99-1.00) (Table 2). Female index clients with at least one partner newly diagnosed with HIV were 1.3 times (RR: 1.29, 95% CI: 1.23-1.36; aRR: 1.25, 95% CI: 1.18-1.32) more likely to have named additional partners. Female index clients with inconsistent condom use (: 0.84, 95% CI: 0.73-0.96) and those who had sex with more than one partner (aRR: 0.85 (0.75-0.96) were less likely to name additional partners.

**Table 2:** Factors associated with female index clients who named additional partners via assisted partner services at follow-up visits, N = 872.

Variable	Univariate		Multivariate	
	RR^a^ (95% CI)	P-value	aRR^b^ (95% CI)	P-value
**Socio-demographic characteristics**				
Age	1.00 (0.99–1.01)	0.417	1.00 (0.99–1.00)	0.083
Marital status				
Single/Never married	Ref	Ref	Ref	Ref
Married (monogamous)/Cohabitating	0.90 (0.77–1.05)	0.173	0.84 (0.72–0.97)	0.022
Married (polygamous)	1.02 (0.84–1.25)	0.824	0.89 (0.73–1.07)	0.206
Divorced/Separated/Widowed	0.95 (0.82–1.09)	0.450	0.85 (0.72–1.00)	0.051
Highest education completed				
Did not complete primary school	Ref	Ref	Ref	Ref
Completed primary school	1.09 (0.97–1.22)	0.148	1.00 (0.89–1.12)	0.996
Completed secondary school	1.06 (0.93–1.21)	0.383	1.05 (0.94–1.18)	0.383
Occupation				
Employed/Self-employed	Ref	Ref	Ref	Ref
Unemployed	1.08 (0.91–1.28)	0.398	1.08 (0.95–1.22)	0.242
Student	1.01 (0.86–1.17)	0.944	0.95 (0.82–1.09)	0.461
Monthly household income >87 USD^b^	0.95 (0.85–1.07)	0.413	1.07 (0.97–1.18)	0.180
Homa Bay County (Ref: Kisumu)	1.66 (1.33–2.05)*	<0.001*	1.25 (0.99–1.00)*	0.023*
**HIV testing/ testing and sexual history**				
Facility based testing	1.25 (1.05–1.48)*	0.012*	1.06 (0.94–1.19)	0.325
Tested as a couple	1.05 (0.83–1.32)	0.709	1.02 (0.68–1.55)	0.830
HIV test prior to study enrollment	1.12 (0.89–1.42)	0.338	0.92 (0.73–1.16)	0.344
HIV self-test in last 12 months	0.70 (0.55–0.90)	0.004	0.85 (0.56–1.30)	0.099
Number of partners in the last 3 years	1.29 (1.23–1.36)*	<0.001*	1.25 (1.18–1.32)*	<0.001*
**Behaviors associated with HIV transmission in the last 12 months**				
Any	0.93 (0.76–1.14)	0.475	1.16 (0.99–1.35)	0.070
Inconsistent condom use	0.88 (0.73–1.07)	0.193	0.84 (0.73–0.96)	0.013*
Did not use condom during last sex	0.95 (0.79–1.15)	0.621	1.00 (0.87–1.15)	0.959
Recent sexually transmitted infection (STI)	0.95 (0.66–1.37)	0.784	0.93 (0.74–1.16)	0.510
Recurrent sex under influence of alcohol/recreational drugs	0.41 (0.20–0.87)	0.019*	0.58 (0.26–1.33)	0.199
Sex with a partner(s) who is HIV positive	1.05 (0.77–1.44)	0.738	1.15 (0.91–1.45)	0.244
Sex with more than one partner	0.81 (0.63–1.05)	0.117	0.85 (0.75–0.96)	0.007*
Sex partner(s) at high risk for HIV or HIV status unknown	0.97 (0.65–1.44)	0.872	1.11 (0.80–1.54)	0.531
**Partner outcomes**				
≥ 1 partners newly diagnosed with HIV	1.44 (1.28–1.61)*	<0.001*	1.24 (1.05–1.48)*	<0.001*

^a^RR: relative risk, ^a^ RR: relative risk adjusted for age, county, testing modality, number of partners in the last 3 years, condom use during last sex, inconsistent condom use, recent STI, sex with >1 partner, sex partner(s) HIV positive, sex partner(s) at high risk for HIV or HIV status unknown, and sex under the influence of drugs.

^b^USD: United States dollar (data was collected in Kenya shillings, with about Kenya shillings 10,000 exchanging for 87 USD).

We did not find statistically significant differences between index participants who did and did not name additional partners in terms of sociodemographic characteristics, HIV testing history/method, or other sexual behaviors that have been associated with HIV transmission.

### Male partner characteristics

Female index clients named 3461 male partners, an average of 4 male partners per female index. Of these partners, 2920 (84%) were notified of their potential exposure via APS and enrolled in the study; 2152 (74%) did not have a prior diagnosis and underwent HIV testing. Of the 2920 partners notified, 1819 (62%) were named initially, of whom 430 (24%) had a prior diagnosis and 1386 (76%) tested for HIV. Of those tested, 90 (6.5%) received a new HIV diagnosis. During follow-up visits, index clients named an additional 1101 (38%) male partners, of whom 766 (64%) tested for HIV and 193 (25%) of these individuals received a new HIV diagnosis.

Male partners named at the initial visit had a median age of 35 years (IQR: 29–40); 1370 (75%) were married monogamously or cohabiting, 1603 (88%) were employed, and 1115 (80%) of those without a prior diagnosis had tested for HIV before study enrollment (Table 3). Male partners who were named later had a median age of 38 (IQR 33–42), 896 (82%) were married monogamously or cohabiting, the majority were employed (1065, 97%), and 699 (91%) of those without a prior diagnosis had previously tested for HIV.

**Table 3:** Characteristics of male partners by initial and additional elicitation at follow-up visits via assisted partner services.

Variable	Initially named male partners N = 1819	Additionally named male partners^b^ N = 1101	Total N = 2920
	n (%) or median (IQR^a^)	n (%) or median (IQR)	n (%) or median (IQR)
**Socio-demographic characteristics**			
Age	35 (29,40)	38 (33,42)	36 (30,41)
Marital status			
Single/Never married	278 (15.3)	84 (7.6)	362 (12.4)
Married (monogamous)/Cohabitating	1370 (75.3)	896 (81.5)	2266 (77.6)
Married (polygamous)	94 (5.2)	64 (5.8)	158 (5.4)
Divorced/Separated/Widowed	77 (4.2)	56 (5.1)	133 (4.6)
Highest education completed			
Did not complete primary school	349 (19.2)	117 (10.6)	466 (16.0)
Completed primary school	739 (40.6)	435 (39.5)	1174 (40.2)
Completed secondary school	731 (40.2)	549 (49.9)	1280 (43.8)
Occupation			
Employed/Self-employed	1603 (88.1)	1065 (96.8)	2668 (91.4)
Unemployed	171 (9.4)	21 (1.9)	192 (6.6)
Student	45 (2.5)	14 (1.3)	59 (2.0)
Monthly household income			
**≤** 87 USD^c^	1146 (63.0)	608 (55.3)	1754 (60.1)
> 87 USD	672 (36.9)	492 (44.7)	1165 (39.9)
County			
Kisumu	848 (46.6)	234 (21.3)	1082 (37.1)
Homa Bay	971 (53.4)	867 (78.7)	1838 (62.9)
**HIV testing/ testing and sexual history**			
Testing modality			
Non-facility based	1229 (67.6)	635 (57.7)	1864 (63.8)
Facility based	590 (32.4)	466 (42.3)	1056 (36.2)
Testing approach^d^			
Individual	1346 (97.1)	761 (99.3)	2107 (97.9)
Couple	40 (2.9)	5 (0.7)	45 (2.1)
HIV test prior to study enrollment	1524 (83.8)	1032 (93.7)	2556 (87.5)
HIV test prior to study enrollment (excluding prior diagnoses)^d^	1115 (80.3)	699 (91.3)	1814 (84.2)
HIV self-test in last 12 months	149 (8.2)	190 (17.3)	339 (11.6)
HIV status at the time of study enrolment			
Negative	1296 (71.2)	573 (52.0)	1869 (64.0)
Known positive	430 (23.6)	335 (30.4)	765 (26.2)
New positive	90 (4.9)	193 (17.5)	283 (9.7)
Unknown	3 (0.2)	0	3 (0.1)
**Behaviors associated with HIV transmission in the last 12 months** ^**d**^			
Inconsistent condom use	429 (31.0)	380 (49.6)	809 (37.6)
Did not use condom during last sex	376 (27.1)	330 (43.1)	706 (32.8)
Recent sexually transmitted infection (STI)	30 (2.2)	44 (5.7)	74 (3.4)
Recurrent use of post-exposure prophylaxis (PEP)	17 (1.2)	29 (3.8)	46 (2.1)
Ever used pre-exposure prophylaxis (PrEP)	13 (0.9)	29 (3.8)	42 (2.0)
Recurrent sex under influence of alcohol/recreational drugs	18 (1.3)	12 (1.6)	30 (1.4)
Sex with more than one partner	261 (18.8)	296 (38.6)	557 (25.9)
Sex partner(s) at high risk for HIV and HIV status unknown	7 (0.5)	13 (1.7)	20 (0.9)
Sex partner(s) HIV positive	105 (7.6)	60 (7.8)	165 (7.7)
Transactional sex	5 (0.4)	2 (0.3)	7 (0.3)
Injection drug use with shared needles/syringes	2 (0.1)	1 (0.1)	3 (0.1)
**Elicitation period**			
0–3 months	1819 (100.0)	262 (23.8)	2081 (71.3)
4–6 months	–	243 (22.1)	243 (8.3)
7–9 months	–	306 (27.8)	306 (10.5)
10–12 months	–	256 (23.3)	256 (8.8)
> 12 months	–	34 (3.1)	34 (1.2)

^a^IQR: interquartile range.

^b^Male partners named by a female index after the first visit (and within 12 months of the index’s diagnosis).

^c^USD: United States dollar (data was collected in Kenya shillings, with about Kenya shillings 10,000 exchanging for 87 USD).

^d^Data only available for 2155 participants without prior diagnosis.

Restricting to those without a prior HIV diagnosis, male partners named at follow-up visits differed from those named at initial visits (Table 4). Male partners named at follow-ups were more commonly from Homa Bay county, slightly older, more likely to be or have been married, and had higher levels of education, employment, and income (Table 4). The association with county, marital status, and education but not age remained significant in the multivariate analysis. Male partners named at follow-up visits also reported more instances of behaviors associated with risk of HIV transmission including: inconsistent condom use (p = 0.002), not using a condom at last sex (p = 0.010), having sex with more than one partner (p < 0.001), having used pre-exposure prophylaxis (p < 0.001), having a recent sexually-transmitted infection (p < 0.001), recurrently using post-exposure prophylaxis (p < 0.001), and having sex with a partner at risk of HIV or with HIV status unknown (p = 0.001). In an multivariate analysis, having sex with more than one partner (p < 0.001) and having sex with a partner at risk of HIV or with HIV status unknown (p = 0.010) remained significantly higher among male partners named after follow up visits.

**Table 4:** Factors associated with male partners who were named at follow-up visits (vs. initially named) via assisted partner services.

Variable	Univariate, partners without prior diagnosis N = 2155		Multivariate, partners without prior diagnosis N = 2155	
	RR (95% CI)	P-value	aRR^a^(95% CI)	P-value
**Socio-demographic characteristics**				
Age	1.02 (1.01–1.03)*	<0.001*	1.01 (1.00–1.02)	0.077
Marital status				
Single/Never married	Ref	Ref	Ref	Ref
Married(monogamous)/Cohabitating	1.70 (1.27–2.28)*	<0.001*	1.37 (1.04–1.79)*	0.024*
Married (polygamous)	2.04 (1.37–3.04)*	<0.001*	1.36 (0.99–1.87)	0.058
Divorced/Separated/Widowed	1.79 (1.11–2.88)*	0.016*	1.43 (1.01–2.02)	0.043
Highest education completed				
Did not complete primary school	Ref	Ref	Ref	Ref
Completed primary school	1.46 (1.12–1.89)*	0.005*	1.27 (1.00–1.61)	0.046*
Completed secondary school	1.67 (1.26–2.21)*	<0.001*	1.3 (0.98–1.72)*	0.068
Occupation				
Employed/Self-employed	Ref	Ref	Ref	Ref
Unemployed	0.29 (0.17–0.48)*	<0.001*	0.46 (0.27–0.79)*	0.004*
Student	0.65 (0.36–1.16)	0.147	1.17 (0.61–2.25)	0.633
Monthly household income >87 USD^b^	1.23 (0.98–1.55)	0.077	1.02 (0.81–1.28)	0.880
Homa Bay county (Ref: Kisumu)	2.22 (1.58–3.10)*	<0.001*	1.47 (1.04–2.08)*	0.029*
**HIV testing/ testing history**				
Facility based testing modality	1.62 (1.11–2.36)*	0.012*	1.43 (1.18–1.75)*	<0.001*
Testing approach - as a couple^c^	0.31 (0.11–0.83)*	0.021*	0.31 (0.11–0.86)*	0.009*
HIV test prior to study enrollment	1.96 (1.44–2.67)*	<0.001*	1.33 (1.06–1.66)*	0.014*
HIV self-test in last 12 months	1.89 (1.56–2.28)*	<0.001*	1.77 (1.45–2.16)*	<0.001*
Any	2.13 (1.38–3.30)*	<0.001*	1.30 (0.83–2.05)	0.256
Inconsistent condom use	1.63 (1.19–2.24)*	0.002*	1.13 (0.92–1.40)	0.243
Did not use condom during last sex	1.55 (1.11–2.16)*	0.010*	1.19 (0.95–1.48)*	0.125
Recent sexually transmitted infection (STI)	1.71 (1.29–2.27)*	<0.001*	1.45 (0.84–2.50)	0.185
Recurrent use of post-exposure prophylaxis (PEP)	1.80 (1.35–2.41)*	<0.001*	0.99 (0.70–1.40)	0.950
Ever used pre-exposure prophylaxis (PrEP)	1.98 (1.48–2.64)*	<0.001*	1.77 (0.96–3.25)	0.067
Recurrent sex under influence of alcohol/recreational drugs	1.13 (0.52–2.42)	0.762	0.87 (0.60–1.27)	0.464
Sex with more than one partner	1.80 (1.30–2.60)*	<0.001*	1.31 (1.15–1.50)*	<0.001*
Sex partner(s) at high risk for HIV or HIV status unknown	1.84 (1.06–3.18)*	0.001*	1.61 (1.12–2.31)	0.010*
Sex with a partner(s) who is HIV positive	1.02 (0.55–1.89)	0.941	0.92 (0.60–1.39)	0.687

^a^aRR: relative risk adjusted for age, county, marital status, occupation, income, prior HIV test, testing approach, testing modality, self-testing history, HIV diagnosis, condom use during last sex, inconsistent condom use, recent STI, sex with >1 partner, sex partner(s) HIV positive, sex partner(s) at high risk for HIV or HIV status unknown, recurrent use of PEP, sex under the influence of drugs, and having ever used PrEP.

^b^USD: United States dollar (data was collected in Kenya shillings, with about Kenya shillings 10,000 exchanging for 87 USD).

^c^Data only available for 2155 participants without prior diagnosis.

Among partners tested, there was a 3.9-times higher likelihood of receiving a new HIV diagnosis for those named during follow-up visits compared to initial visits (RR: 3.88, 95% CI: 3.00–4.98). The association between being named during follow-up visits and receiving a new HIV diagnosis remained significant after adjusting for age, county, marital status, occupation, income, prior HIV test, testing approach, testing modality, self-testing history, and transmission-associated behaviors (aRR: 4.82, 95% CI: 2.67-6.36).

The highest frequency of new diagnoses occurred among partners named at a follow-up visit within the first 3 months, with 91 (45%) of 203 partners without a prior diagnosis receiving a positive test result (S1 Table). This is almost double the next highest elicitation period (24% among those named in months 10–12) and almost 7-fold higher than for initially named partners (6.5%). In a multivariate analysis restricted to partners named during follow-up visits, those who were newly diagnosed with HIV were more likely than those who tested negative to be older (p = 0.006), tested at a facility (p = 0.039), and used a HIV self-test in the year prior to being notified for APS (p = 0.018) and had recurrent sex under influence of alcohol/recreational drugs (p = 0.018) and were less likely to be elicited between 4 and 9 months (Table 5).

**Table 5:** Factors associated with new HIV diagnosis (vs. testing negative) among male partners elicited during assisted partner services follow-up visits, N = 766.

Variable	Univariate		Multivariate	
	RR^a^ (95% CI)	P-value	aRR^b^ (95% CI)	P-value
**Socio-demographic characteristics**				
Age	1.03 (1.01–1.04)*	<0.001*	1.02 (1.00–1.04)*	0.006*
Marital status				
Single/Never married	Ref	Ref	Ref	Ref
Married (monogamous)/Cohabitating	1.09 (0.69–1.74)	0.710	1.01 (0.61–1.66)	0.968
Married (polygamous)	1.78 (0.98–3.23)	0.059	1.42 (0.83–2.42)	0.197
Divorced/Separated/Widowed	1.86 (1.10–3.18)	0.022	1.53 (0.87–2.70)	0.141
Highest education completed				
Did not complete primary school	Ref	Ref	Ref	Ref
Completed primary school	0.68 (0.43–1.07)	0.092	0.82 (0.59–1.16)	0.265
Completed secondary school	0.60 (0.35–1.04)	0.068	0.71 (0.49–1.05)	0.087
Occupation				
Employed/Self-employed	Ref	Ref	Ref	Ref
Unemployed	1.40 (0.77–2.54)	0.266	1.20 (0.62–2.33)	0.583
Student	0.61 (0.15–2.57)	0.502	0.72 (0.14–3.61)	0.687
Monthly household income >87 USD^c^	1.27 (0.69–2.33)	0.439	1.16 (0.85–1.58)	0.348
Homa Bay county (Ref: Kisumu)	0.57 (0.39–1.04)*	0.002*	0.79 (0.55–1.12)	0.179
**HIV testing/ testing history**				
Facility based testing modality	0.50 (0.32–0.78)*	0.002*	0.59 (0.36–0.97)*	0.039*
Testing approach - as a couple	2.40 (1.77–3.27)	<0.001	–	–
HIV test prior to study enrollment	0.56 (0.37–0.86)*	0.008*	0.84 (0.53–1.34)	0.468
HIV self-test in last 12 months	0.42 (0.28–0.63)*	<0.001*	0.56 (0.35–0.90)*	0.018*
**Behaviors associated with risk of HIV transmission in the last 12 months**				
Any	0.66 (0.39–1.11)*	0.115	0.76 (0.40–1.43)*	0.390
Inconsistent condom use	1.03 (0.44–2.37)	0.951	1.11 (0.69–1.77)	0.673
Did not use condom during last sex	0.72 (0.44–1.17)*	0.185	0.92 (0.70–1.22)	0.565
Recent sexually transmitted infection (STI)	0.71 (0.40–1.27)	0.250	0.91 (0.44–1.90)	0.805
Ever used pre-exposure prophylaxis (PrEP)	0.40 (0.20–0.82)	0.013*	0.64 (0.24–1.71)	0.375
Recurrent use of post-exposure prophylaxis (PEP)	0.40 (0.21–0.76)	0.005	–	–
Recurrent sex under influence of alcohol/recreational drugs	2.02 (0.98–4.13)	0.055	1.70 (1.10–2.63)*	0.018*
Sex with more than one partner	0.94 (0.38–2.38)	0.904	1.16 (0.65–2.08)	0.610
Sex partner(s) at high risk for HIV or HIV status unknown	1.54 (0.65–3.63)	0.323	1.61 (0.96–2.69)	0.072
Sex with a partner(s) who is HIV positive	0.85 (0.38–1.91)	0.693	1.44 (0.84–2.47)	0.187
**Period when additionally elicited**				
0–3 months	Ref	Ref	Ref	Ref
4–6 months	0.36 (0.22–0.58)*	<0.001*	0.51 (0.29–0.89)*	0.018*
7–9 months	0.33 (0.20–0.56)*	<0.001*	0.45 (0.29–0.70)*	<0.001*
10–12 months	0.53 (0.28–1.04)*	0.063	0.68 (0.44–1.04)	0.075
> 12 months	0.39 (0.14–1.12)*	0.081	0.51 (0.24–1.09)	0.083

^a^RR: relative risk.

^b^aRR: relative risk adjusted for age, county, marital status, education, income, testing modality, self-testing history, prior HIV test, condom use during last sex, inconsistent condom use, recent STI, sex with >1 partner, sex partner(s) HIV positive, sex partner(s) at high risk for HIV or HIV status unknown, recurrent use of PEP, sex under the influence of drugs, and having ever used PrEP.

^c^USD: United States dollar (data was collected in Kenya shillings, with about Kenya shillings 10,000 exchanging for 87 USD).

## Discussion

This study demonstrates that additional partner elicitation increased the efficiency and reach of the APS program in Western Kenya. Specifically, we found that asking women to name male partners up to 12 months after the initial visit identified male partners at high risk of HIV and resulted in a 4-fold increase in new HIV diagnosis in these partners as compared to those initially named. Male partners who were named by index clients after the initial contact with index clients, reported more behaviors associated with HIV transmission such as having sex with more than one partner and having sex with a partner at risk of HIV or with HIV status unknown. This strongly suggests a benefit to continuing to offer APS to women at follow-up visits after a new HIV diagnosis and to providing HIV testing to the male partners they name during these visits.

Additional elicitation increased the reach to partners who would not have otherwise been elicited at the initial visit, e.g., the visit immediately after receiving a new HIV diagnosis. Women experience stigma associated with reporting multiple sex partners, which is compounded by a lack of trust or established rapport with the HTS counsellor and by not being emotionally ready to disclose details of their intimate relationships [12–14]. Fear of loss of income, desertion by primary partner, or risk of emotional or physical violence from the primary partner upon HIV status disclosure have been reported in several studies as a source of hesitancy to identify partners [15,16]. In support of this, a Tanzanian study found that participants were more likely to name more partners if they did not fear rejection, were aware of notification methods and confidentiality protections, and had privacy during HTS [17]. Index clients may need more time to process a new HIV diagnosis before being ready to name partners [18], and changes in an individual’s circumstances may also influence their willingness to discuss partner notification [4]. The 2016 WHO Guidelines on HIV self-testing and partner notification highlighted the issue of trust in health providers in the implementation considerations for success of APS [4]. Continued elicitation after the initial visit is one strategy that allows for building rapport and overcoming some of these barriers to elicitation. Another strategy that has improved partner elicitation in Nigeria is an elicitation box which allows index clients to report sexual contacts on paper and insert in a box for a health care provider to contact at a later time [19]. Additional research is needed to better understand strategies to improve partner elicitation at initial visits and during follow-up for HIV care.

Additionally-named male partners reported more sexual behaviors associated with HIV transmission in the last 12 months. The former included having sex with more than one partner and having sex with a partner at risk of HIV or with unknown HIV status. Current APS programs focus on eliciting partners during the initial visit and have shown that index clients, especially women, with casual partners and those at high risk of HIV maybe less able or willing to name them [20]. This would be consistent with studies that have found that the decision to provide information about a partner is affected by the type of relationship, and that index clients are most willing to name partners they live with, who are presumably part of a stable relationship [15,16]. Interestingly, female index clients who were at higher risk (inconsistent condom use and sex with more than one partner) were not as likely to name additional partners. While it is unclear why this maybe, continued elicitation may be useful in identifying additional partners that are unnamed at initial visit and those that are new to women post-diagnosis. It will be necessary to assure women that they can safely and confidentially name partners so that their partners test, mutually disclose HIV status and use prevention strategies.

Our study found a 4-fold increase in new HIV diagnosis among partners elicited after the initial visit over 12 months of follow up. New diagnoses were most common among partners elicited in the first 3 months after the initial visit. While our study supports the continuation of APS after the initial visit, more research is needed to inform recommendations on the timeframe within which APS can be most impactful. Following a new HIV diagnosis, care is transitioned from the HIV testing point to the HIV clinics for initiation of antiretroviral therapy. Coordination will be required between HTS providers at HIV testing points, adherence counselors, peer educators, and clinicians providing HIV care. There would also need to be training to provide uniform implementation across service delivery points. Prioritizing index clients who are most likely to have male partners with undiagnosed HIV would help reduce complexity and reduce resources required to implement continued elicitation.

Our study has several limitations. We recruited only female index clients, and therefore, cannot determine whether there is a benefit to asking male index clients to name female partners during the 12 months following diagnosis. The study was also limited to western Kenya and may not be generalizable to other settings. The covariates included in the multivariable model were only measured at enrollment and not at each elicitation and this limits assessment individual-level changes in index client characteristics over time, i.e., between initial elicitation at diagnosis and additional elicitation over 12 months. Future qualitative research on why index participants did not name certain partners at the initial encounter with their HTS provider will help us understand and identify barriers to partner elicitation.

## Conclussion

Providing APS to female index clients for 12 months after their initial diagnosis reached partners with more to report sexual behaviors associated with risk of HIV transmission and substantially increased the number of new HIV diagnoses among partners. APS programs should work with HIV care providers to develop strategies to provide ongoing APS and avoid missed opportunities in the identification of partners with HIV. It is important that APS providers build trust with index clients to identify all partners, particularly those who may have a higher risk of acquiring HIV or have undiagnosed HIV.

## Supporting information

S1 Table Test positivity among partners without a prior diagnosis by elicitation time period. (DOCX)

S1 Checklist Inclusivity in global research. (DOCX)
