# Supplementary material for: Improving HIV assisted partner services outcomes by eliciting additional partners after the initial encounter
Source: PLOS Glob Public Health. 2026 Feb 3;6(2):e0004406. doi: 10.1371/journal.pgph.0004406 (PMC12867224; doi:10.1371/journal.pgph.0004406)
Supplement: S1 Table — (DOCX) [file pgph.0004406.s001.docx]

| **Period when additionally elicited** | **Visit named** | **Total** | **New diagnoses**  **N (%)** |
| --- | --- | --- | --- |
| 0-3 months | initial | 1386 | 90 (6.5) |
| 0-3 months | follow-up | 203 | 91 (44.8) |
| 4-6 months | follow-up | 162 | 26 (16.0) |
| 7-9 months | follow-up | 200 | 30 (15.0) |
| 10-12 months | follow-up | 167 | 40 (24.0) |
| >12 months | follow-up | 34 | 6 (17.6) |

**S1 Table. Test positivity among partners without a prior diagnosis by elicitation time period**
